# An overlapping set of genes is regulated by both NFIB and the glucocorticoid receptor during lung maturation

**DOI:** 10.1186/1471-2164-15-231

**Published:** 2014-03-25

**Authors:** Mathieu Lajoie, Yu-Chih Hsu, Richard M Gronostajski, Timothy L Bailey

**Affiliations:** 1 Institute for Molecular Bioscience, The University of Queensland, 306 Carmody Road, St Lucia 4072, Australia; 2 Department of Biochemistry and Developmental Genomics Group, University at Buffalo, 701 Ellicott St., 14203 Buffalo, USA

**Keywords:** Lung development, Nr3c1, Glucocorticoid receptor, Nfib, Regulation of transcription, ChIP-seq analysis, Expression analysis, Motif analysis, Transcription factor

## Abstract

**Background:**

Lung maturation is a late fetal developmental event in both mice and humans. Because of this, lung immaturity is a serious problem in premature infants. Disruption of genes for either the glucocorticoid receptor (*Nr3c1*) or the NFIB transcription factors results in perinatal lethality due to lung immaturity. In both knockouts, the phenotype includes excess cell proliferation, failure of saccularization and reduced expression of markers of epithelial differentiation. This similarity suggests that the two genes may co-regulate a specific set of genes essential for lung maturation.

**Results:**

We analyzed the roles of these two transcription factors in regulating transcription using ChIP-seq data for NFIB, and RNA expression data and motif analysis for both. Our new ChIP-seq data for NFIB in lung at E16.5 shows that NFIB binds to a NFI motif. This motif is over-represented in the promoters of genes that are under-expressed in *Nfib*-KO mice at E18.5, suggesting an activator role for NFIB. Using available microarray data from *Nr3c1-KO* mice, we further identified 52 genes that are under-expressed in both *Nfib* and *Nr3c1* knockouts, an overlap which is 13.1 times larger than what would be expected by chance. Finally, we looked for enrichment of 738 recently published transcription factor motifs in the promoters of these putative target genes and found that the NFIB and glucocorticoid receptor motifs were among the most enriched, suggesting that a subset of these genes may be directly activated by *Nfib* and *Nr3c1*.

**Conclusions:**

Our data provide the first evidence for *Nfib* and *Nr3c1* co-regulating genes related to lung maturation. They also establish that the *in vivo* DNA-binding specificity of NFIB is the same as previously seen *in vitro*, and highly similar to that of the other NFI-family members NFIA, NFIC and NFIX.

## Background

Lung development is a complex developmental process initiated by budding of the lungs from the gut endodermal tube, multiple rounds of expansion and branching morphogenesis, and final maturation of the epithelial and endothelial components that comprise the airways, pulmonary circulation, and gas exchange surface [1,2]. It is the final maturation of the lung epithelial cells that is frequently interrupted by premature birth, leading to both acute and chronic lung disease in premature infants [3,4]. Here we demonstrate apparently related roles of the *Nfib* and glucocorticoid receptor (hereafter either *Nr3c1* or GR) genes in lung maturation.

Previous studies showed that loss of *Nfib* resulted in perinatal lethality due to lung immaturity [5]. The lungs of late fetal mice lacking *Nfib* showed reduced expression of Type I and Type II epithelial markers along with morphological immaturity exemplified by a failure of the formation of saccules, the precursor to the alveolar air exchange region. In addition, excess proliferation of both mesenchymal and epithelial cells is seen in *Nfib* null lungs. Surprisingly, while the phenotype is clearly related to the failure of epithelial cell maturation, loss of *Nfib* only in the mesenchymal cells of the lung yields a very similar phenotype [6], indicating that mesenchymal cells regulate late epithelial maturation through as yet unknown inductive mechanisms [7].

Prenatal administration of glucocorticoids has been shown to stimulate lung maturation in both mice and premature infants [8-10]. Conversely, deletion of *Nr3c1*, the gene encoding the glucocorticoid receptor, results in a phenotype remarkably similar to that of loss of *Nfib*, including excess cell proliferation, failure of saccularization and reduced expression of markers of epithelial differentiation [10]. As with *Nfib*, loss of *Nr3c1* only in the mesenchyme recapitulates much of this phenotype [11]. The similarity in phenotype seen with the loss of either *Nfib* or *Nr3c1*, together with the shared cell-type expression requirement suggests that these two genes may co-regulate a specific set of genes essential for lung maturation. We therefore examined the lung genes regulated by *Nfib* and *Nr3c1* and the specific binding targets of NFIB to determine how these genes may cooperate in the regulation of lung maturation.

## Results

### ChIP-seq shows that NFIB binds to the known NFI motif in mouse fetal lung

**Figure 1:**
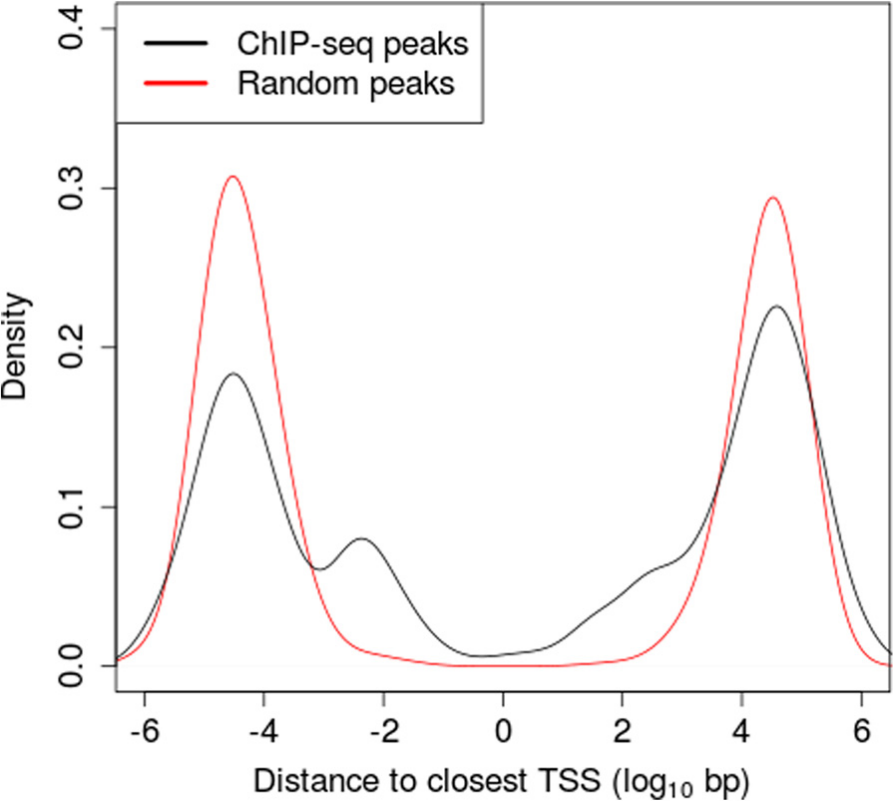
**Distribution of NFIB ChIP-seq peaks relative to closest TSS.** The black curve represents the distribution of the distances between the 759 NFIB ChIP-seq peaks and the closest TSS. Negative distances correspond to upstream peaks and positive distances to downstream peaks. The red curve shows the distances when the peaks are randomly and uniformly repositioned on their chromosomes. Density is estimated using a Gaussian kernel with bandwidth *h*=0.4.

We conducted a ChIP-seq analysis of NFIB in wild type mouse fetal lung at E16.5 and identified 759 peaks from an initial set of 8,717,818 unpaired reads (see Methods). The distribution of the distances between these peaks and the closest TSS shows a strong enrichment within 1 kbp both upstream and downstream of the TSSs compared to a random control (Figure 1). Peaks are particularly enriched at about 100bp upstream of the nearest known TSS, showing that NFIB frequently binds the proximal promoter. There is also considerable enrichment of peaks downstream of the nearest known TSS for several hundred base-pairs. This could represent either binding in the 5’UTR of the known gene or binding in the promoter of an unannotated alternative transcript.

**Figure 2:**
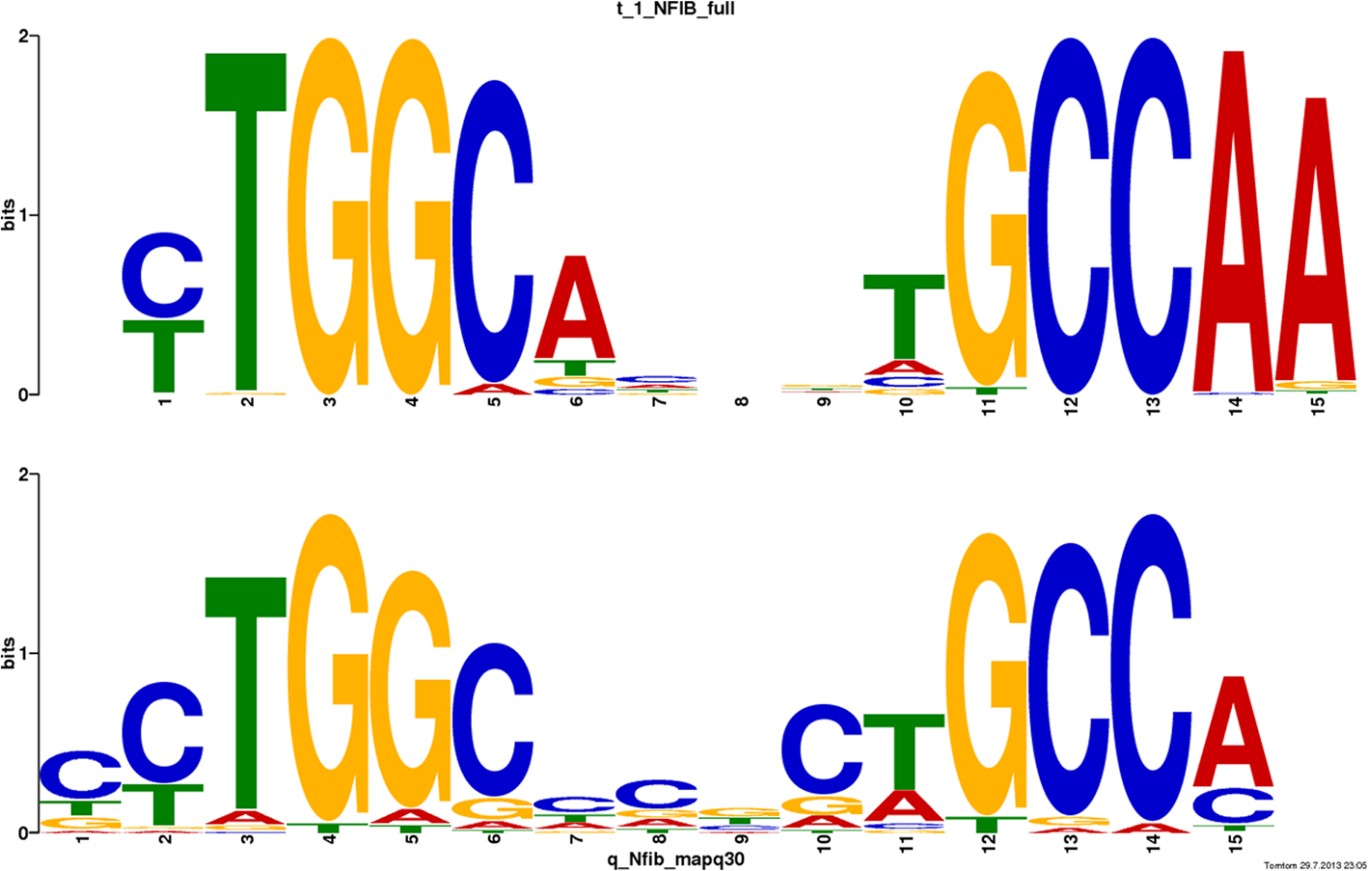
**NFIB DNA-binding motifs in mouse fetal lung and *in vitro*.** The motif reported by Jolma *et al. *[14] using SELEX is shown above the motif discovered by MEME in mouse fetal lung NFIB ChIP-seq peak regions.

We applied the MEME algorithm [12] to repeat-masked [13], 100bp genomic regions centered on each of the 759 NFIB ChIP-seq peaks. The most statistically significant motif found by MEME matches the known NFIB palindromic consensus sequence TGGCnnnnnGCCA. More importantly, the motif found by MEME is extremely similar to the *in vitro* NFIB motif obtained by Jolma *et al.*[14] using SELEX technology (Figure 2). This observation confirms that NFIB has similar DNA-binding specificity in mouse fetal lung cells as in a cell-free *in vitro* system. The palindromic binding motif found by MEME further strongly suggests that NFIB binds mainly as a dimer in these cells. Finally, the strong similarity between the *in vivo* and *in vitro* motifs for NFIB in Figure 2 show that the ChIP-seq experiment and downstream data analysis succeeded.

**Figure 3:**
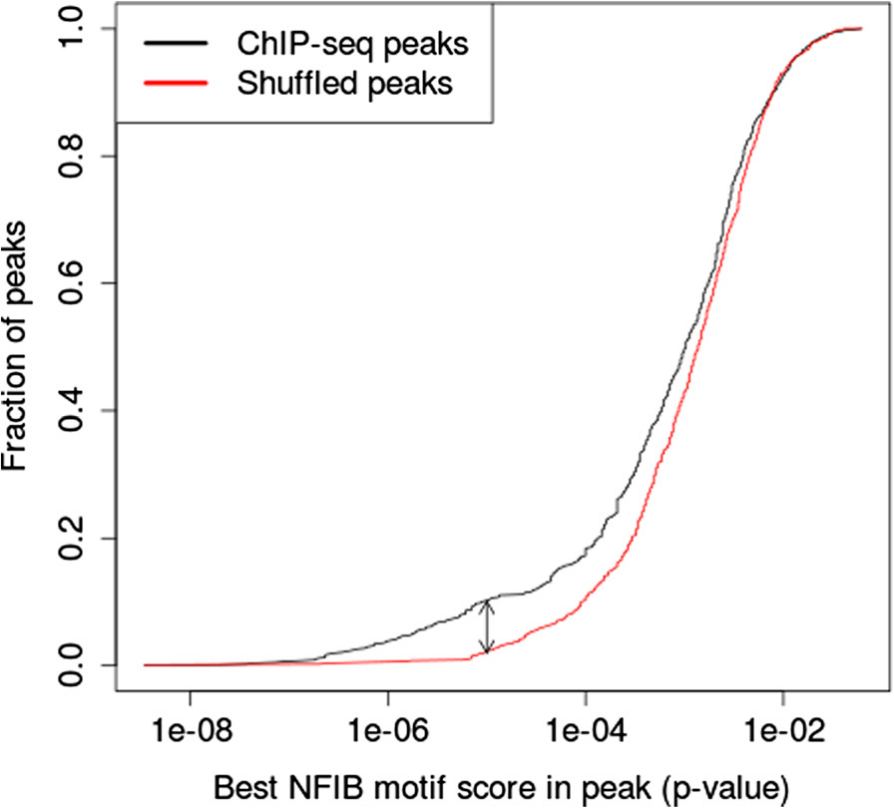
**Enrichment of the NFIB motif in NFIB ChIP-seq peaks.** The black curve shows the fraction of 759 ChIP-seq peaks with at least one predicted NFIB binding site using different motif score thresholds. The red curve shows distribution obtained when each ChIP-seq peak region is shuffled while preserving trinucleotide frequencies.

To further assess the quality of our ChIP-seq data, we considered the fraction of predicted ChIP-seq peaks that contain a match to the discovered NFIB motif at different motif score thresholds, as computed by the FIMO scanning algorithm [15]. At a motif score *p*-value threshold of 10^-5^, the NFIB motif is present in 10.1% of the ChIP-seq peaks but in only 1.7% of the randomized control sequences (Figure 3). This represents a six-fold enrichment (77/13 = 5.92), which exceeds the ENCODE guidelines requiring at least 10% of the peaks to have a four-fold enrichment for the ChIPed TF’s binding motif [16].

### Correlation of NFIB binding and expression of nearby genes

We studied the mechanism of transcriptional regulation by NFIB in fetal lung cells using our NFIB ChIP-seq data from E16.5 fetal lung cells and previously published gene expression data from E18.5 fetal lung cells in WT and *Nfib*-knockout mice [6].

We first sought for dysregulated genes in the *Nfib*-KO using a 2-fold expression change threshold and a maximal *q*-value of 0.05 for selection (see Methods).

We identified 631 genes, of which 412 are down-regulated and 219 are up-regulated. For convenience, we will refer to the down-regulated genes as “NFIB-activated”, and to the up-regulated as “NFIB-repressed”. Of course, we realize that the observed effect could be due to direct or indirect regulation of the gene in question (e.g., via NFIB interacting with another TF).

We then counted the numbers of genes with an NFIB ChIP-seq peak within 1 kbp, 10 kbp or 100 kbp (Table 1). As can be seen in Table 1, only 0.3% of the NFIB-activated genes (1 gene) have an NFIB ChIP-seq peak within 1 kbp of their TSS. This is a lower percentage than for all genes (1.2%), but the difference is not statistically significant (*p*=0.1, two-tailed Fisher’s exact test).

**Table 1:** Number of NFIB-activated/repressed genes with an NFIB ChIP-seq peak near their TSS

	**≤1 kbp**	**≤10 kbp**	**≤100 kbp**
Activated (396)	0.3% (1)	1.3% (5)	10.6% (42)
Repressed (203)	1.0% (2)	2.0% (4)	8.4% (17)
All (11383)	1.2% (140)	2.2% (255)	11.6% (1324)

The table shows the percentage (number) of NFIB-activated, NFIB-repressed or all genes that have an NFIB peak within the given distance of their TSS. Percentages are in terms of the total number of genes in the given class. Note that we consider only genes for which we have both distance and expression data, so the numbers of activated/repressed genes presented in this table are slightly lower than the total number of NFIB-activated genes (412) and NFIB-repressed genes (219).

If we extend the analysis to binding at up to 10 kbp and 100 kbp from the TSS, neither the NFIB-activated nor NFIB-repressed genes have a number of NFIB ChIP-seq peaks that differs significantly from the number expected by chance (*p*>0.05, two-tailed Fisher’s exact test).

The lack of evidence of a clear relationship between proximal NFIB binding and gene expression in Table 1 may be due to the fact that the expression data is from a later stage of fetal lung development than the ChIP-seq data (E18.5 vs. E16.5). It is quite possible that the set of genes bound by NFIB changes substantially between E16.5 and E18.5. Another confounding factor is that the gene expression data comes from embryonic lungs where *Nfib* is deleted from E10, but expression is not measured until E18.5, leaving ample time for compensatory changes in gene expression. In fact among the 631 genes identified as activated or repressed at day E18.5 in the *Nfib*-KO mouse, 28 are annotated as having “sequence-specific DNA binding transcription factor activity” in the Gene Ontology (GO) database [17]. The changes in expression of these TFs will affect the expression of many genes, so many of the observed dysregulated genes may be *indirect* rather than direct targets of *Nfib*. Another possibility is that the majority of regulation by NFIB is via long-distance chromatin looping [18], but we consider this unlikely given the clear enrichment of NFIB binding events we observe in proximal promoter regions (Figure 1).

### Promoters of genes activated by NFIB are enriched in NFIB motifs, but repressed ones are not

In the absence of ChIP-seq data in E18.5 mouse fetal lung, we turned to a motif-based analysis of the relationship between NFIB binding and gene expression. First, we tested for over-representation of putative NFIB binding sites (predicted using our new NFIB motif) in the promoters of NFIB-activated and NFIB-repressed genes (see Methods). We found a significant enrichment in the NFIB-activated genes (*p*_*act*_<0.0013), but not in the NFIB-repressed (*p*_*rep*_>0.3). This suggest that many genes are activated by NFIB through direct interaction, but that repression generally results from indirect regulation.

**Figure 4:**
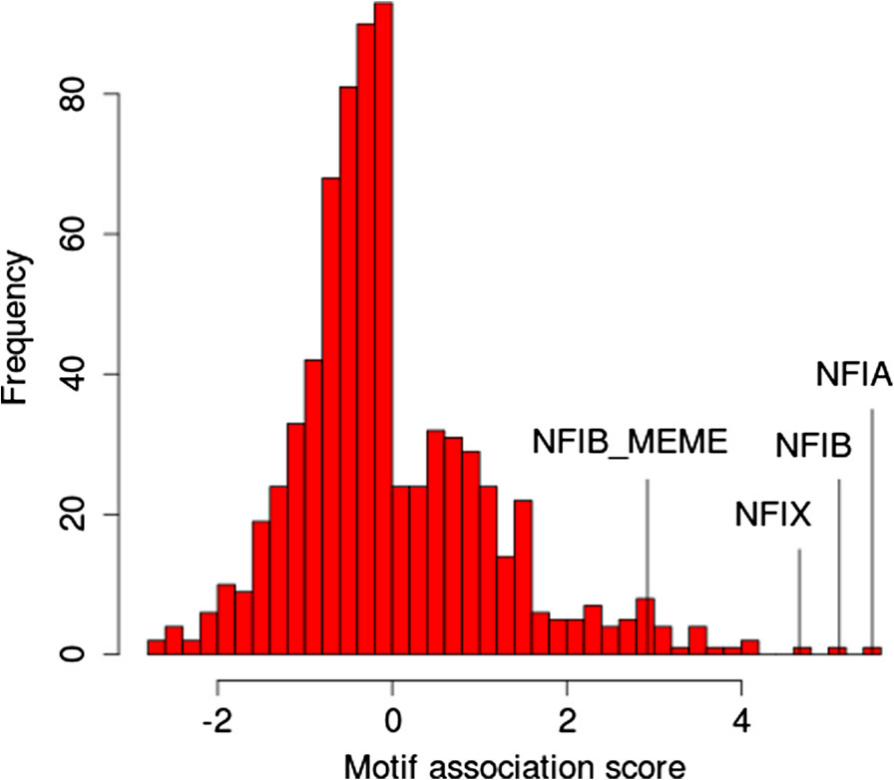
**Motif Association Score distribution for *Nfib *targets.** The histogram shows the distribution of the MAS for the 739 motifs in the compendium (our ChIP-seq motif inferred with MEME (Nfib_MEME) plus 738 mouse and human TF motifs determined by SELEX [14]).

As a control and to get a broader picture, we then calculated *p*_*act*_ and *p*_*rep*_ and our motif association score (MAS, see Methods) for each of the 738 SELEX-derived motifs reported by Jolma *et al.*[14], which cover the DNA-binding specificity of most transcription factor families in mammals. We found that the three motifs with the largest MAS are the three NFI-family motifs in the Jolma *et al.*[14] compendium (Figure 4 and Table 2). These motifs have large *positive* MAS scores, which indicates that their presence in the promoter of a gene is highly correlated with it having *reduced* expression in the *Nfib*-KO mouse at E18.5. This suggests that NFIB acts as a direct *activator* of transcription for many genes in our NFIB-activated set, in mouse fetal lung at E18.5. The complete MAS results are given in Additional file 1.

**Table 2:** **Association of TF motifs with *Nfib *** **targets**

***Motif***	***Gene***	***FC***	***p***_***a*** ***c*** ***t***_	***p***_***r*** ***e*** ***p***_	**MAS**
NFIA_full	*Nfia*	2.27	<10^-5^	0.31	5.50
NFIB_full	*Nfib*	*2.75	<10^-5^	0.27	5.12
NFIX_full	*Nfix*	-	<10^-4^	0.26	4.67
Meis3_DBD_2	*Meis3*	NA	<10^-4^	0.44	4.13
MEIS3_DBD_2	*Meis3*	NA	<10^-4^	0.44	4.03
Meis2_DBD_2	*Meis2*	1.57	0.0001	0.53	3.98
SNAI2_DBD	*Snai2*	1.72	0.0002	0.86	3.78
Rxra_DBD	*Rxra*	-	0.0004	0.54	3.49
EBF1_full	*Ebf1*	-	0.0004	0.46	3.49
Rxrb_DBD	*Rxrb*	NA	0.0004	0.69	3.49
NR2F6_DBD_2	*Nr2f6*	-	0.0004	0.75	3.46
RXRA_DBD	*Rxra*	-	0.0006	0.62	3.22
NR2F1_DBD	*Nr2f1*	1.83	0.0007	0.74	3.18
RXRB_DBD	*Rxrb*	NA	0.0007	0.41	3.16
Pknox2_DBD	*Pknox2*	-	0.0009	0.41	3.05
TFAP4_full	*Tfap4*	NA	0.0010	0.24	3.02
PKNOX2_DBD	*Pknox2*	-	0.0010	0.60	3.00
RXRG_DBD	*Rxrg*	NA	0.0011	0.49	2.96
NR2C2_DBD	*Nr2c2*	-	0.0012	0.65	2.93
Nfib_MEME	*Nfib*	*2.75	0.0012	0.34	2.92

The table shows the TF motif name (motif), associated gene name (gene), expression fold-change (FC) of the gene, the (unadjusted) motif enrichment *p*-value in the activated set of genes (*p*_*act*_), the (unadjusted) motif enrichment *p*-value in the repressed set of genes (*p*_*rep*_), and the motif association score (MAS). Rank is based on the magnitude of the MAS, “NA” indicates that no expression data is available for the TF gene and “-” in the FC column indicates that expression change is not significant (*p*-value >0.05). Motif names all in uppercase are derived from human TFs, others are from mouse TFs. *Although *Nfib* expression increases in the *Nfib*-KO, no functional protein is produced.

As an additional control, we repeated the complete MAS analysis after replacing mouse promoter sequences by their ortholog from rat or human. For both species, NFI motifs were among the most strongly enriched within the set of NFIB-activated genes. In human, NFIX ranked second (*p*_*act*_<10^-4^), and NFIB ranked fourteenth (*p*_*act*_<10^-3^). In rat, NFIX ranked third (*p*_*act*_<10^-3^) and NFIB ranked tenth (*p*_*act*_<10^-3^). Overall, this strongly support the hypothesis that NFIB activates its targets during lung maturation through direct interactions near the promoter regions.

The presence of our novel, ChIP-derived NFIB motif in gene promoters shows less significant correlation with gene expression than the SELEX-based motifs for NFIA, NFIB and NFIX (Table 2). The new motif (NFIB_MEME) ranks twentieth according to MAS, despite being highly similar to the SELEX-based NFIB motif (Figure 2), from which it differs primarily in the preference for an ‘A’ in the right-most position. This may indicate that the new, ChIP-based motif is slightly less accurate than the SELEX-based motif, which, if true, could be due to numerous reasons. The accuracy of motifs derived from ChIP-seq experiments depends strongly on the number of sequences without the motif presented to the motif discovery algorithm. Such sequences can be due to imperfect antibody specificity or to indirect DNA-binding by the antibody via a protein complex or via chromatin loops bound jointly by the antibody and another DNA-binding protein [19]. None of these issues are present in SELEX experiments, although they suffer from their own limitations. There is no guarantee that the DNA-binding specificity of the protein or DNA-binding domain assayed by SELEX is the same under the *in vitro* SELEX conditions as it is in *in vivo*. At any rate, our expression correlation results suggest that the existing SELEX-based NFI-family motifs are at least as accurate as our ChIP-derived motif for NFIB.

**Figure 5:**
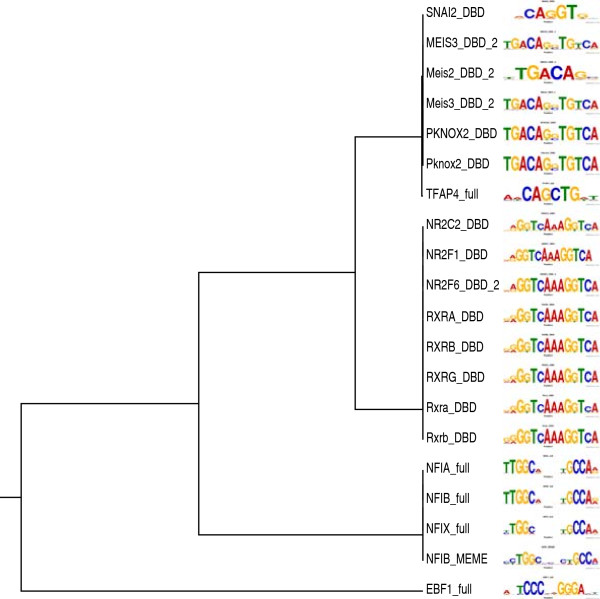
**Motifs associated with dysregulated genes in *Nfib*-KO mouse clustered by motif similarity.** The tree shows the top 20 motifs according to MAS clustered using the Pearson Correlation Coefficient to measure the similarity between pairs of motifs, and the UPGMA tree-building algorithm to create the tree [20]. The tree was drawn using Phylodendron [21].

Although our new NFIB motif ranks twentieth among the 739 motifs in the combined motif database, there are effectively only three distinct motifs with higher MAS scores (Figure 5). This is because the compendium contains motifs for both mouse and human TFs (e.g., Meis3 and MEIS3) and it contains multiple members of transcription factor families. Paralogous and orthologous TFs tend to have highly similar DNA-binding affinities, as shown by the highly similar motif logos at the leaves of the motif tree in Figure 5.

### Other TFs may contribute to the *Nfib*-KO phenotype

For each motif in Table 2 we have included the expression fold change of the corresponding gene, if significant (*p*-value ≤0.05). The increased expression of *Nfia* in the *Nfib*-KO further suggests that there may be some compensatory mechanism at play between the two paralogs. Since the DNA-binding motif of NFIA is almost identical to that of NFIB, it is probable that both transcription factors bind to the same regulatory elements. Such an apparently compensatory change in one NFI family member upon loss of another was noted previously in *Nfib*-KO lungs and suggests some type of homeostatic regulation of total NFI levels [5]. However, these data are not sufficient to indicate whether NFIA acts as an activator or a repressor, or whether the same genes are regulated by both NFIA and NFIB.

In addition to the NFI motifs, we note the large positive MAS of the Meis, SNAI2 and NR2F1 motifs, indicating that they are enriched in the NFIB-activated gene set (i.e. in genes that are under-expressed in the *Nfib*-KO). Because *Snai2*, *Meis2* and *Nr2f1* are over-expressed in the *Nfib*-KO, it is possible that some genes in our NFIB-activated set are repressed by these genes instead of being directly activated by NFIB. Repressor activity has been documented for each of these three factors [22-24]. For example *Snai2*, which represses transcription via the recruitment of histone deacetylases to target gene promoters [25], is known for its antiapoptotic activity and plays a role in epithelial-mesenchymal transition. While neither epithelial-mesenchymal transition nor altered apoptosis seem implicated in the phenotype of *Nfib*^-/-^ lungs [6], other epigenetic changes mediated by *Snai2* affecting cell proliferation and cell differentiation are clearly possible and will be investigated. Finally, we note the enrichment of the EBF1 motif, which regulates cell differentiation [26]. However, according to the microarray data, the *Ebf1* gene is not significantly dysregulated in the *Nfib*-KO.

The apparently paradoxical increased expression of *Nfib* in the *Nfib*-KO lungs (Table 2) is explained by the fact that only exon 2 (containing the DNA-binding domain and dimerization domain) is actually knocked out. The microarray is detecting an increase in a transcript that is missing exon 2, and thus cannot lead to a functional NFIB protein. One explanation for this increased expression from the *Nfib* promoter in the absence of production of a functional *Nfib* transcript is that NFIB normally represses its own production, either directly or indirectly (Table 3). A second possibility is that the shorter, disrupted transcript is less subject to post-transcriptional degradation that the complete transcript, leading to higher measured expression in the *Nfib*-KO lungs.

**Table 3:** **Comparison of expression change for some TFs of interest in *Nfib*- and *Nr3c1*-knockout lungs**

	***Nfib*-KO**		***Nr3c1*-KO**	
***Gene***	**FC**	***p*-*value***	**FC**	***p*-*value***
*Nfib*	*2.75	<10^-5^	2.17	0.001
*Nr3c1*	1.23	0.172	*3.14	<0.001
*Nfia*	2.27	0.001	0.61	0.006
*Snai2*	1.72	<10^-5^	1.54	0.003
*Meis2*	1.57	0.023	2.03	0.012
*Nr2f1*	1.83	0.001	1.66	0.003

For each knockout, the table shows the expression fold change and the associated uncorrected *p*-value (see Methods). The expression changes for *Nfib* and *Nr3c1* transcripts in their respective KO (marked by an asterisk) occurs in the context of the transcripts lacking an essential exon and thus no functional protein is expressed.

### *Nfib* and *Nr3c1* regulate an overlapping set of genes

*Nfib*-knockout mice show a phenotype very similar to that seen in glucocorticoid receptor (*Nr3c1*) knockout mice. To ask whether this may be due to a common set of dysregulated genes, we compared our *Nfib*-KO microarray data with available microarray data for *Nr3c1*-KO in fetal mice lung at 18.5 [27]. Using the same selection threshold as for the *Nfib*-KO dataset (2-fold expression change and *q*-value ≤ 0.05), we identified 158 activated genes and 160 repressed genes by GR.

The sets of genes activated or repressed by GR overlap significantly with the analogous gene sets for NFIB (Table 4). The sets of activated genes share 52 genes in common, which is 13.1 times higher than what would be expected by chance, while the sets of repressed genes have 22 genes in common, a 9-fold enrichment.

**Table 4:** **Regulatory targets of *Nfib *** **and *Nr3c1 *** **overlap significantly**

	***Activated***	***Repressed***
*Nfib*	340	171
*Nr3c1*	117	143
Observed overlap	52	22
Expected overlap	4.0	2.5
Overlap fold enrichment	13.1	9.0
Overlap enrichment *p*-value	<10^-46^	<10^-15^

The table shows the sizes of the sets of activated and repressed genes for NFIB and GR, the size of the overlaps of the gene sets, the expected overlap size, fold enrichment of the overlap and the *p*-value of the overlap enrichment. Note that to compute the enrichment *p*-value, we must consider only the genes for which we have expression data for both knockouts (9990 genes), so the numbers of activated/repressed genes in this table are different than the numbers reported for the individual knockouts.

**Figure 6:**
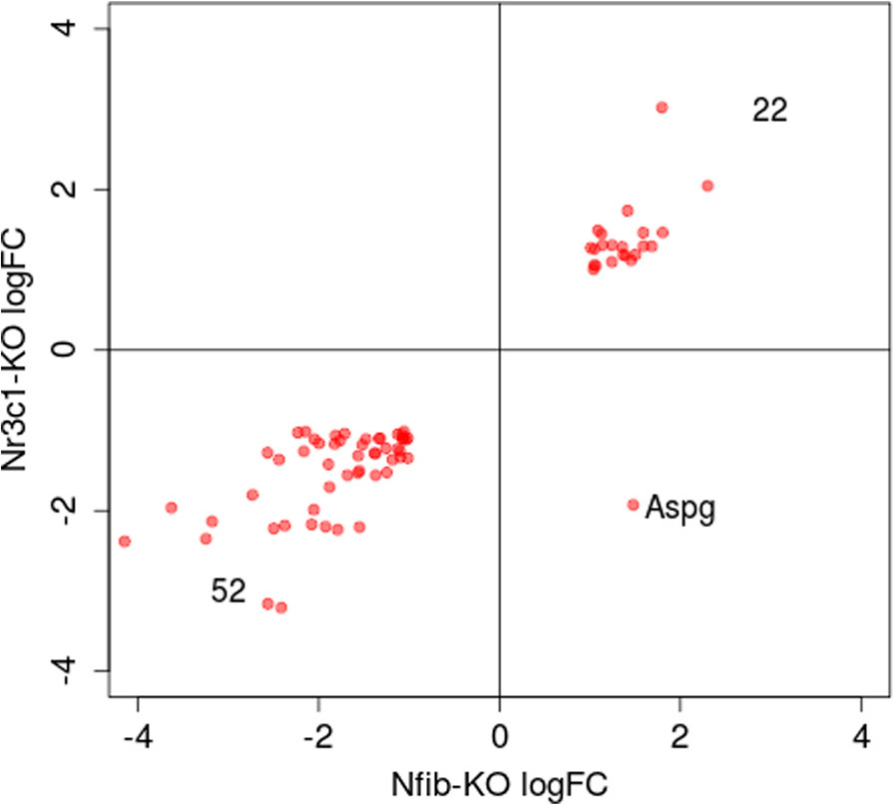
**Expression change of genes activated/repressed in both *Nfib*-KO and *Nr3c1*-KO mice is highly correlated.** Each point represents a gene activated/repressed in *both* knockouts. Expression change is expressed as log2(KO/WT), and only genes with a 2-fold change and a *q*-value less than 0.05 in both knockouts are shown.

As we can see from Figure 6, the direction of the change in expression is the same for all but one of the genes activated/repressed in the two knockout experiments (Figure 6). The one gene whose expression changes in a different direction is Aspg (log2(FC) = 1.48 in *Nfib*-KO; -1.93 in *Nr3c1*-KO). The Pearson correlation coefficient between the log2(FC) in the two knockouts of these genes is 0.907. This strongly suggests that the common phenotype in the two knockouts are due to some or all of the genes in the overlap set. The list of genes activated/repressed in both datasets is available in Additional file 2.

### Promoters of genes that are activated by both NFIB and GR are enriched in NFIB and GR motifs

**Figure 7:**
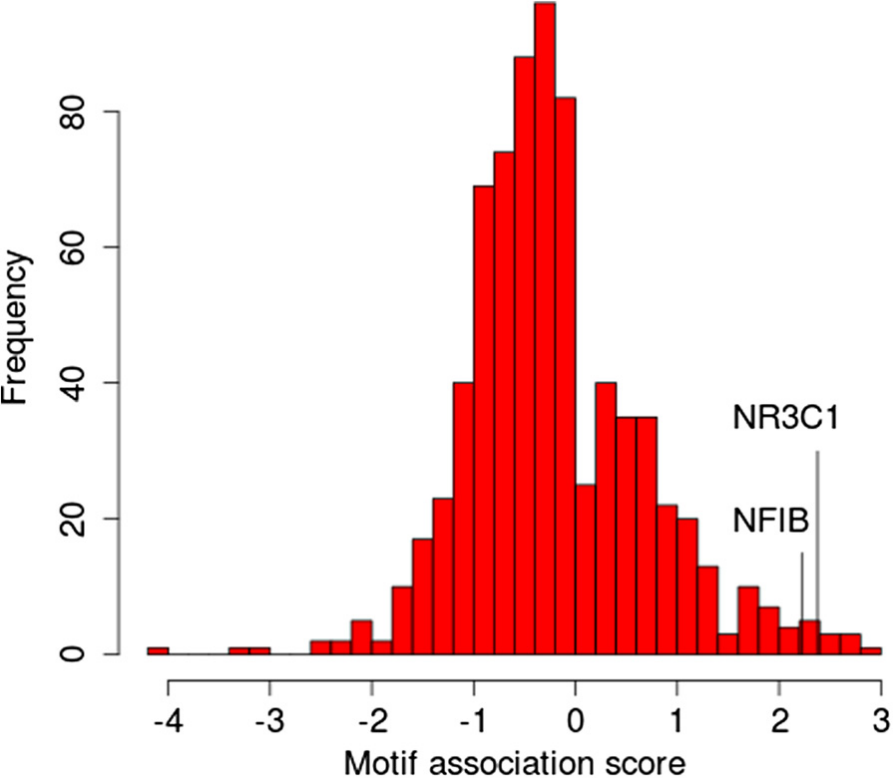
**Motif Association Score distribution for *Nfib *and *Nr3c1 *targets.** The histogram shows the distribution of the MAS for the 739 motifs in the compendium (our ChIP-seq motif inferred with MEME (Nfib_MEME) plus 738 mouse and human TF motifs determined by SELEX [14]).

To further test our hypothesis that *Nfib* and *Nr3c1* co-regulate an overlapping set of genes, we looked for motif enrichment in the sets of commonly activated or repressed genes identified above. Figure 7 shows the distribution of the MAS score for 739 motifs in the Jolma *et al.*[14] compendium, and the relative position of the GR (NR3C1) and NFIB motifs. We note that the MAS score of the two motifs are highly significant and among the largest positive ones, ranking at the 8th and 12th positions, respectively (Table 5). The complete results are available in Additional file 3.

**Table 5:** **Association of TF motifs with *Nfib *** **and *Nr3c1 *** **common targets**

***Rank***	***Motif***	***Gene***	**FC**_***N*** ***f*** ***i*** ***b***_	**FC**_***N*** ***r*** **3** ***c*** **1**_	***p***_***a*** ***c*** ***t***_	***p***_***r*** ***e*** ***p***_	**MAS**
1	AR_full	*Ar*	-	-	0.0013	0.97	2.86
2	TBR1_full	*Tbr1*	NA	-	0.0016	0.19	2.79
3	EBF1_full	*Ebf1*	-	-	0.0018	0.89	2.75
4	TBX2_full_2	*Tbx2*	-	-	0.0020	0.37	2.71
5	ZNF410_DBD	*Zfp410*	1.24	-	0.0025	0.90	2.60
6	NR3C2_DBD	*Nr3c2*	-	-	0.0036	0.82	2.44
7	Ar_DBD	*Ar*	-	-	0.0038	0.93	2.42
8	NR3C1_DBD	*Nr3c1*	-	*3.14	0.0042	0.84	2.38
9	TBX1_DBD	*Tbx1*	-	-	0.0048	0.01	2.32
10	AR_DBD	*Ar*	-	-	0.0054	0.75	2.26
11	EOMES_DBD	*Eomes*	NA	-	0.0057	0.14	2.24
12	NFIB_full	*Nfib*	*2.75	2.17	0.0060	0.13	2.22
...	...	...	...	...	...	...	...
732	ESRRB_DBD	*Esrrb*	NA	1.31	0.10	0.0071	-2.15
733	MEF2A_DBD	*Mef2a*	0.72	-	0.91	0.0060	-2.22
734	POU3F1_DBD_2	*Pouf3f1*	NA	NA	0.97	0.0050	-2.30
735	ESRRG_full_3	*Esrrg*	-	-	0.14	0.0038	-2.42
736	OTX1_DBD	*Otx1*	NA	NA	0.78	0.0036	-2.44
737	Esrra_DBD_2	*Esrra*	NA	NA	0.03	0.0008	-3.10
738	ESRRA_DBD	*Esrra*	NA	NA	0.13	0.0005	-3.26
739	FOXI1_full_2	*Foxi1*	NA	-	0.62	<10^-4^	-4.01

The table shows the rank of the MAS score (rank), the motif name (motif), the associated gene name (gene), the expression fold-change (FC) of the gene in each KO, the (unadjusted) motif enrichment *p*-value in the activated set of genes (*p*_*act*_), the (unadjusted) motif enrichment *p*-value in the repressed set of genes (*p*_*rep*_) and the motif association score (MAS). “NA” indicates that no expression data is available for the TF gene and “-” indicates that expression change is not significant (*p*-value >0.05). Motif names all in uppercase are for human TFs, others are for mouse TFs. *Note that these FC apply to the disrupted transcripts, and that no functional protein is produced for *Nfib* and *Nr3c1*.

Among the motifs with a larger (but similar) positive MAS, we find the androgen receptor (which is almost identical to GR), the EBFI motif, motifs for two members of the T-box family of transcription factors and the ZNF410 motif. However, none of the mouse genes corresponding to these motifs shows a significant (*p*- value ≤0.05) expression change in both the *Nr3c1* and *Nfib* knockouts. We also note the large *negative* MAS of *Foxi1* and some estrogen-related receptors (*Esrra*, *Esrrb*). Estrogen controls many cellular processes such as growth and differentiation. While *Esrrb* is over-expressed in the *Nr3c1*-KO, we identified no such dysregulation in the *Nfib*-KO. We have no expression data for *Foxi1* in *Nfib*-KO, and this gene is not dysregulated in the *Nr3c1*-KO. However, some other genes of the same Fox family are dysregulated in both knockouts, such as *Foxp2* (over-expressed in both) and *Foxn3* (under-expressed in *Nr3c1*-KO and over-expressed in *Nfib*-KO). Interestingly, it has been shown that loss of *Foxp2* leads to defective postnatal lung alveolarization in mouse [28].

Similarly to what we did in a previous section, we repeated the MAS analysis using human and rat orthologous sequences. For human, NFIX ranked third (*p*_*act*_<10^-4^), and NFIB ranked nineteenth (*p*_*act*_<10^-2^). For rat, NFIX ranked sixth (*p*_*act*_<10^-3^), and NFIB ranked eighth (*p*_*act*_<10^-2^). This suggests that NFIB activates some NR3C1-activated genes through binding at their promoter sequences. However, NR3C1 did not show a significant enrichment for human, and it ranked only 51 ^st^ in rat (*p*_*act*_=0.044). These data suggest that in some instances mechanisms other than direct binding of promoter sequences by NR3C1 may mediate co-regulation by NR3C1 and NFIB. Consistent with this finding, previous studies have indicated that some functions of NR3C1 are mediated by mechanisms other than direct NR3C1 binding to DNA. For example, mice defective in DNA-binding by NR3C1 are viable while those deleted for NR3C1 die at birth [29]. Thus it will be important to determine the fraction of co-regulated genes whose expression is regulated by direct binding of NR3C1 versus other indirect mechanisms of regulation.

### Regulatory sub-network involving *Nfib* and *Nr3c1*

**Figure 8:**
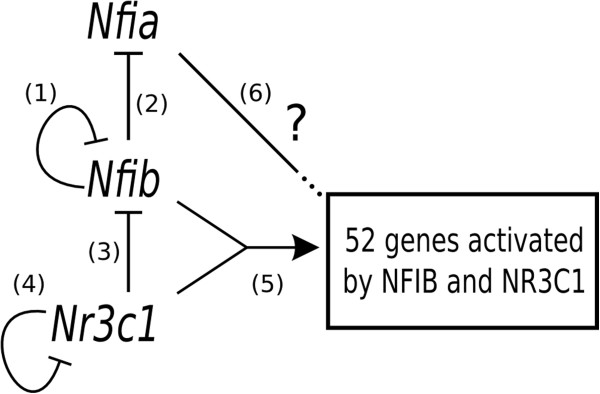
**Possible regulatory sub-network involving *Nfib*, *Nfia *and *Nr3c1*.** A flat head on an edge indicates a repressive effect. The connector labeled 5 indicates cooperative activation by both *Nfib* and *Nr3c1*. Evidence for each numbered edge is given in the main text. The 52 *activated* genes are 2-fold over-expressed in the wildtypes compared to *Nfib*-KO and *Nfib*-KO, with a *q*-value ≤0.05.

Based on the data presented above, we propose a possible regulatory sub-network involving *Nfib*, *Nr3c1*, *Nfia* and their 52 common activated target genes (Figure 8). Each of the links represents activation (pointed arrow) or repression (flat arrow) of transcription by a transcription factor. Each of the links in the figure is numbered, and we describe the experimental support for each link in what follows.

Firstly, as shown in Table 2 and discussed above, *Nfib* and *Nfia* transcripts are both repressed by NFIB, which is indicated by links 1 and 2 in Figure 8. For simplicity, and because both NFIA and NFIB are transcription factors and thus can act directly to affect transcription, we depict these as direct interactions, but they may well be indirect. The repression of *Nfia* by *Nfib* is further supported by the observation that *Nfia* is significantly under-expressed (*p*<10^-5^) while *Nfib* is significantly over-expressed (*p*=0.001) in the *Nr3c1*-KO (Table 3).

Secondly, both *Nr3c1* and *Nfib* transcripts are significantly repressed by GR (*p* < 0.001 and *p* = 0.001, respectively, Table 3), which we indicate by links 3 and 4 in Figure 8. We once again depict these as direct interactions for simplicity.

Thirdly, we propose that the set of 52 genes under-expressed in both knockouts (hereafter G52) are activated cooperatively by *Nfib* and *Nr3c1*. While there may be some indirect regulation at play, our motif analysis revealed a significant enrichment for both NFIB and GR motifs in this set, which suggests that some of these genes are direct targets of these factors. Moreover, the two alternative *trivial* topologies that could connect *Nfib* and *Nr3c1* to G52 are not supported by the data. Indeed, the topology

Nr3c1→Nfib→G52

is precluded by the data supporting edge 3, and the topology

Nfib→Nr3c1→G52

would involve under-expression of *Nr3c1* in the *Nfib*-KO, which is not observed. We indicate the direct cooperation hypothesis by link 5 in Figure 8.

Finally, since the DNA binding affinities of NFIA and NFIB are highly similar (see motif logos in Figure 5), we infer that NFIA may bind many of the same regulatory elements as NFIB. We therefore hypothesize that *Nfia* may regulate the genes in G52, which we indicate by link 6 Figure 8. However, it is not clear from the available data whether *Nfia* acts as a repressor or an activator of these genes.

### GO analysis of putative common targets of *Nfib* and *Nr3c1*

To identify which biological processes could be activated by *Nfib* and *Nr3c1*, we tested for gene-annotation enrichment using GOrilla [30] and a ranked list approach (see Methods). We found significant enrichment for several general terms including “cell adhesion” (q-value = 0.03), “transport” (q-value = 0.0006) and “immune system process” (q-value = 0.004), but this required the inclusion of genes that are not in the set of 52 putative common targets (i.e. genes that appear under-expressed in both knockouts, but that do not meet our strict fold-change and q-value thresholds, see Methods). This suggests that both *Nfib* and *Nr3c1* may regulate these processes in some manner during lung development. On the other hand, we found significant enrichment for three specific terms where *Nfib* and *Nr3c1* are more likely to play a direct role since the associated genes are among the 52 common targets we have identified: First, “cellular defense response” (q-value = 0.03), with the *Tnfrsf4* (tumor necrosis factor receptor superfamily, member 4) and *Ncf1* (neutrophil cytosolic factor 1) genes; Second, “regulation of vascular endothelial growth factor signaling pathway” (q-value = 0.02), with the *Myoc1c* (myosin ic) and *Xdh* (xanthine dehydrogenase) genes; Third, “regulation of fibroblast proliferation” (q-value = 0.04), with *Sphk1* (sphingosine kinase 1), *Aqp1* (aquaporin 1) and *Cdkn1a* (cyclin-dependent kinase inhibitor 1a) genes. We are currently examining these genes in more detail to assess NFIB and GR binding to putative regulatory regions.

## Discussion

Lung maturation is a complex process dependent on cooperation between the mesenchyme, epithelium and endothelial cells to promote vascularization, epithelial differentiation, mesenchyme thinning and lung morphogenesis [1,2]. Previous studies using transcriptional profiling have identified large numbers of genes whose expression levels change substantially during each of the characteristic stages of mouse lung development: 1) embryonic, 2) pseudoglandular, 3) canalicular, 4) saccular and 5) alveolar [31-33]. For example Mariani *et al.* demonstrated groups of extracellular matrix genes that exhibit stage-specific expression patterns in mouse lung [32,33]. However the vast majority of these changes in gene expression represent stage-specific differentiation markers of epithelial or mesenchymal cells which define the cellular phenotype at each stage, but give little information on the regulatory mechanisms that control the differentiation process. Conversely, genetic studies have been instrumental in the discovery of gene regulatory pathways essential for early, and to a lesser extent, later stages of lung development. For example, reciprocal epithelial-mesenchymal inductions have been shown to be essential for both early and late stages of lung development [7,34,35]. In addition, while a number of signaling pathways including the FGF [36,37], Shh [38,39], BMP [40,41], Wnt [42] and TGF [43,44] pathways are known to signal within and between the epithelium and mesenchyme, the transcriptional networks that respond to and/or generate these pathways and mediate the maturation program remain largely unknown [2,45].

Both the NFIB and GR transcription factors have been shown previously to regulate the transition from the canalicular to the saccular stage in mouse lung [5,10,46,47]. Indeed mutations in either gene result in a very similar phenotype characterized by an excess of mesenchymal and epithelial cells at E18, severe reduction or failure of saccule formation, severe delay in type I and type II cell differentiation and resultant perinatal lethality with largely non-inflated lungs. More recently it was determined using conditional KO alleles that *Nfib* and *Nr3c1* expression in the lung mesenchyme, not lung epithelium, is essential for normal lung maturation [6,11]. The similarity in phenotype seen with loss of either *Nfib* or *Nr3c1* initially suggested that these two transcription factors might be cooperating in a conserved pathway of lung maturation. Our determination of a significant overlap between the genes whose expression changes with the loss of either *Nfib* or *Nr3c1* (Table 4 and Figure 6) in lung mesenchyme is consistent with this hypothesis. In addition, the motif analysis showed significant enrichment of NFI and GR binding motifs in genes whose expression decreased in both *Nfib*-KO and *Nr3c1*-KO lungs (Figure 7) suggesting cooperative activation of these genes by these factors.

## Conclusions

Our computational analysis combining expression, binding and motif data provide the first evidence for *Nfib* and *Nr3c1* co-regulating genes related to lung maturation. Although these data are consistent with the model of *Nfib* and *Nr3c1* function shown in Figure 8, other models are possible. For example, it may be that the overlap in differentially expressed genes in the two mutants (Table 4) is a reflection of the overall phenotype (lung immaturity) rather than being causally dependent on direct co-regulation by *Nfib* and *Nr3c1*. To distinguish between such alternate hypotheses it will be necessary to simultaneous assess GR and NFIB binding to mouse lung target genes and assess the frequency of co-occupancy at potential target genes. While GR ChIP-seq data is available for human lung cancer cells [48], rat pheochromocytoma cells [49] and mouse adipocytes [50], because GR binding to DNA is highly dependent on chromatin context and thus shows high cell-type specificity [51] these data sets are of marginal use at best for determining GR targets in normal lung mesenchyme. In addition, while the ChIP-seq data set used here for NFIB was determined at E16.5, it will be necessary to assess both NFIB and GR binding at multiple stages of lung development to determine the temporal sequence during which NFIB and GR bind to target sites to regulate gene expression. However, even in the absence of such data our determination that both NFIB and GR binding motifs are among those most highly associated with genes whose expression is decreased by loss of either *Nfib* and *Nr3c1* (Figure 8) provides a useful framework for future studies.

## Methods

### NFIB ChIP-seq

E16.5 wild type lungs were minced on ice in PBS, incubated in 1% formaldehyde at room temperature for 10 min., quenched with 0.125M glycine for 5 min. and prepared for chromatin isolation as described previously [6]. Chromatin was sheared to ∼200–500 bp using a Branson Sonifier 250 sonicator and subjected to ChIP using a ChIP assay kit (Upstate Biotechnology) and NFIB antibody (Geneka Biotechnology). Immunoprecipitated chromatin was isolated, the crosslinks reversed, and the isolated DNA was used to prepare a sequencing library and sequenced at the Cornell University DNA Sequencing and Genotyping Laboratory, resulting in 8,717,818 unpaired reads of 42bp.

Reads were aligned to the mouse genome (mm9) using Bowtie2 software [52] with default parameters. 2,311,332 reads (26.51%) aligned at a unique location and 971,349 (11.14%) aligned to more than one location. Reads with a mapping quality score (MAPQ) below 30 were discarded. The remaining reads were then used to call peaks with the MACS1.4 software [53], using default parameter values except for the following: -g 1.87e9 (effective genome size) -m 5,30 (MFOLD parameter). The list of peak summits output by MACS is available in Additional file 4. *De novo* motif discovery was performed on the 100bp sequences centered around each peak summit with repeats masked (mm9, downloaded from UCSC), using the MEME software [12]. DNA sequences were shuffled using the uShuffle software [54] with parameter k=3 to preserve trinucleotide frequencies.

### Microarray analysis

For *Nfib*-KO, we used the mRNA expression profiling datasets published in [6]. In this KO strain only exon 2 is deleted from the *Nfib* gene leaving the remaining exons present for detection in the microarray analysis. These datasets have been produced using Affymetrix arrays and are available at the NCBI Gene Expression Omnibus (GEO) data repository under Accession number GSE24465. The probe intensity signals were normalized using the GC-RMA algorithm.

For *Nr3c1*-KO, we used the datasets published in [27]. These datasets have been produced using Codelink BioArrays and are available at the ArrayExpress data repository under Accession number E-MEXP-861. The signal of the probes with “Good quality” flag was extracted, log2-transformed and quantile normalized using R.

For both datasets, differential expression and *p*-values were calculated using the linear model implemented in the R Limma package (Smyth, 2004), and *q*-values were obtained from the *p*-values using the Benjamini-Hochberg method (they are referred to as *adjusted p-values* in the Limma output). For downstream analysis, we considered only the probe set with the most significant result for each gene. The complete results with all probes are available in Additional files 5 and 6. Note that differential expression is expressed as log2(KO/WT) in Additional files 5 and 6, but are simply expressed as ratio (KO/WT) in the main text.

In this paper we will refer to a gene as *activated* by TF *X* if its expression decreases at least 2-fold in the knockout of *X*, with a *q*-value ≤ 0.05. Similarly, we will say that a gene is *repressed* by *X* if its expression increases at least 2-fold in the KO of *X*, with a *q*-value ≤ 0.05. These two definitions define three sets of genes: 

• *X*-activated genes: *KO*/*WT*≤ 0.5 and *q*-value ≤ 0.05,

• *X*-repressed genes: *KO*/*WT*≥ 2 and *q*-value ≤ 0.05, and

• *X*-non-target genes: All other genes for which we have expression data.

Of course we are using the terms “activated” and “repressed” somewhat loosely here, since these sets of genes will include both direct and indirect regulatory targets of TF *X*.

We use *q*-values in the above definition to take multiple testing into account. However, when we refer to a particular gene that has been selected using another criteria (e.g. a motif analysis), we report unadjusted *p*-values of differential expression, and we say that this gene is dysregulated if its *p*-value ≤ 0.05, without enforcing any fold change threshold.

### Motif enrichment in promoters of target genes

To determine how a given motif *M* is associated with the regulatory targets of TF *X*, we measure the enrichment of *M* in the proximal promoters of genes activated or repressed by X, relative to the promoters of non-target genes. (Note that the motif *M* can be any motif, not just that of the TF *X*).

First, we use motif *M* to scan the proximal promoter of each gene, defined as the region within 1000 bp of its TSS, and save the best score for each promoter. We then determine *p*_*act*_, the *p*-value of a Wilcoxon rank-sum test [55] with the null hypothesis that the promoter motif scores are no better in the activated genes compared to the non-target genes. Similarly, *p*_*rep*_ is the *p*-value when we test the scores of the repressed genes compared with the non-target genes. For analysis and plotting purposes, we combine these two *p*-values into a single motif association score (MAS), whose magnitude is log10(min(*p*_*act*_,*p*_*rep*_)), and whose sign is positive if the motif *M* is more significantly enriched in the activated genes, *p*_*act*_≤*p*_*rep*_, and negative otherwise.

In the current work, we first compute the MAS for *Nfib* targets using each of the 738 motifs in the compendium of SELEX-based motifs published by Jolma *et al.*[14]. In a subsequent analysis, we compute the MAS for the common targets of *Nfib* and *Nr3c1*. In this case, the “activated” set of genes is simply the intersection of the *Nfib*-activated and *Nr3c1*-activated, likewise for the “repressed” set of genes. The set of “non-targets” is the intersection of the *Nfib*-non-targets and the *Nr3c1*-non-targets. We note that since *p*_*act*_ and *p*_*rep*_ are not adjusted for multiple tests, MAS should be viewed as a score rather than as a true statistical confidence measure.

We extracted TSS coordinates from the UCSC browser KnownGene table. When a gene has multiple TSSs, we used the one corresponding to the shortest transcript.

### Intersection between *Nfib* and *Nr3c1* targets

The size of the overlap *z* between two independent sets *A* and *B*, each sampled without replacement from a set *C*, follows a hypergeometric distribution with parameters *m*=|*A*|, *n*=|*C*|-|*A*| and *k*=|*B*|, where |*X*| denotes the number of elements in set *X*. If *C* is the set of all genes, and *A* and *B* are two sets of target genes, the probability that their intersection contains *z* or more genes (under the null model) can be obtained in the R programming language with a call to the hypergeometric distribution function phyper(z-1,m,n,k,lower.tail=FALSE). The expected size of the overlap is equal to *mk*/(*n*+*m*).

### GO-term analysis

All genes were ranked according to their likeliness of being activated by both *Nfib* and *Nr3c1*, with the most likely at the top of the list. To achieve this, we used the maximum of the fold-change (KO/WT) observed in either the *Nfib*-KO or the *Nr3c1*-KO as a sorting key. This ranked list of genes was used as input to GOrilla with default parameters [30], and a q-value threshold of 0.05 was used to define significant results.

## Competing interests

The authors declare that they have no competing interests.

## Authors’ contributions

ML designed and performed the bioinformatics analysis, interpreted the results and drafted the manuscript. YCH performed the ChIP-seq. RMG proposed the study and contributed to the manuscript. TLB supervised the computational study and contributed to the manuscript. All authors read and approved the final manuscript.

## Supplementary Material

Additional file 1 **MAS results for NFIB targets.** Complete MAS results for the 738 Jolma *et al.*[14] motifs + NFIB-MEME motif. Click here for file

Additional file 2 **Expression data for NFIB and NR3C1 common targets.** Expression data for genes that are activated/repressed in both NFIB-KO and NR3C1-KO. Click here for file

Additional file 3 **MAS results for NFIB and NR3C1 common targets.** Complete MAS results for the 738 Jolma *et al.*[14] motifs + NFIB-MEME motif. Click here for file

Additional file 4 **NFIB peaks summits.** Peak summit coordinates in BED format. Click here for file

Additional file 5 **NFIB-KO Microarray Analysis.** File produced with the R Limma package, in TSV format. Click here for file

Additional file 6 **NR3C1-KO Microarray Analysis.** File produced with the R Limma package, in TSV format. Click here for file

## References

[B1] Maeda Y Davé V Whitsett JA **Transcriptional control of lung morphogenesis** Physiol Rev 2007 15 1 219 244 10.1152/physrev.00028.2006 17237346

[B2] Morrisey EE Hogan BL **Preparing for the first breath: genetic and cellular mechanisms in lung development** Dev Cell 2010 15 1 8 23 10.1016/j.devcel.2009.12.010 20152174 PMC3736813

[B3] Popova AP **Mechanisms of bronchopulmonary dysplasia** J Cell Commun Signal 2013 15 2 119 10.1007/s12079-013-0190-x 23334556 PMC3660689

[B4] Roos AB Berg T Nord M **A relationship between epithelial maturation, bronchopulmonary dysplasia, and chronic obstructive pulmonary disease** Pulm Med 2012 15 2 119 10.1155/2012/196194 PMC3540891 23320163

[B5] Steele-Perkins G Plachez C Butz KG Yang G Bachurski CJ Kinsman SL Litwack ED Richards LJ Gronostajski RM **The transcription factor gene Nfib is essential for both lung maturation and brain development** Mol Cell Biol 2005 15 2 685 698 10.1128/MCB.25.2.685-698.2005 15632069 PMC543431

[B6] Hsu Y-C Osinski J Campbell CE Litwack ED Wang D Liu S Bachurski CJ Gronostajski RM **Mesenchymal nuclear factor IB regulates cell proliferation and epithelial differentiation during lung maturation** Dev Biol 2011 15 2 242 252 10.1016/j.ydbio.2011.04.002 21513708 PMC3098902

[B7] Shannon JM Hyatt BA **Epithelial-mesenchymal interactions in the developing lung** Annu Rev Physiol 2004 15 625 645 10.1146/annurev.physiol.66.032102.135749 14977416

[B8] Seckl JR **Prenatal glucocorticoids and long-term programming** Eur J Endocrinol 2004 15 49 62 10.1530/eje.0.151u049 15554887

[B9] Banks BA Cnaan A Morgan MA Parer JT Merrill JD Ballard PL Ballard RA **Multiple courses of antenatal corticosteroids and outcome of premature neonates** Am J Obstet Gynecol 1999 15 3 709 717 10.1016/S0002-9378(99)70517-X 10486488

[B10] Cole TJ Blendy JA Monaghan AP Krieglstein K Schmid W Aguzzi A Fantuzzi G Hummler E Unsicker K Schütz G **Targeted disruption of the glucocorticoid receptor gene blocks adrenergic chromaffin cell development and severely retards lung maturation** Genes Dev 1995 15 13 1608 1621 10.1101/gad.9.13.1608 7628695

[B11] Habermehl D Parkitna JR Kaden S Brügger B Wieland F Gröne H-J Schütz G **Glucocorticoid activity during lung maturation is essential in mesenchymal and less in alveolar epithelial cells** Mol Endocrinol 2011 15 8 1280 1288 10.1210/me.2009-0380 21659474 PMC5417239

[B12] Bailey TL Elkan C **Fitting a mixture model by expectation maximization to discover motifs in biopolymers** Proc Int Conf Intell Syst Mol Biol 1994 15 28 36 7584402

[B13] Smit A **RepeatMasker.** 1996 2010 http://www.repeatmasker.org

[B14] Jolma A Yan J Whitington T Toivonen J Nitta KR Rastas P Morgunova E Enge M Taipale M Wei G Palin K Vaquerizas JM Vincentelli R Luscombe NM Hughes TR Lemaire P Ukkonen E Kivioja T Taipale J **DNA-binding specificities of human transcription factors** Cell 2013 15 1–2 327 339 23332764 10.1016/j.cell.2012.12.009

[B15] Grant CE Bailey TL Noble WS **FIMO: scanning for occurrences of a given motif** Bioinformatics 2011 15 7 1017 1018 10.1093/bioinformatics/btr064 21330290 PMC3065696

[B16] Landt SG Marinov GK Kundaje A Kheradpour P Pauli F Batzoglou S Bernstein BE Bickel P Brown JB Cayting P Chen Y DeSalvo G Epstein C Fisher-Aylor KI Euskirchen G Gerstein M Gertz J Hartemink AJ Hoffman MM Iyer VR Jung YL Karmakar S Kellis M Kharchenko PV Li Q Liu T Liu XS Ma L Milosavljevic A Myers RM **ChIP-seq guidelines and practices of the ENCODE and modENCODE consortia** Genome Res 2012 15 9 1813 1831 10.1101/gr.136184.111 22955991 PMC3431496

[B17] Ashburner M Ball CA Blake JA Botstein D Butler H Cherry JM Davis AP Dolinski K Dwight SS Eppig JT Harris MA Hill DP Issel-Tarver L Kasarskis A Lewis S Matese JC Richardson JE Ringwald M Rubin GM Sherlock G **Gene Ontology: tool for the unification of biology. The Gene Ontology Consortium** Nat Genet 2000 15 1 25 29 10.1038/75556 10802651 PMC3037419

[B18] Kadauke S Blobel GA **Chromatin loops in gene regulation** Biochimica et Biophysica Acta (BBA)-Gene Regulatory Mechanisms 2009 15 1 17 25 10.1016/j.bbagrm.2008.07.002 PMC2638769 18675948

[B19] Farnham PJ **Insights from genomic profiling of transcription factors** Nat Rev Genet 2009 15 9 605 616 10.1038/nrg2636 19668247 PMC2846386

[B20] Mahony S Auron PE Benos PV **DNA familial binding profiles made easy: comparison of various motif alignment and clustering strategies** PLoS Comput Biol 2007 15 3 61 10.1371/journal.pcbi.0030061 PMC1848003 17397256

[B21] Gilbert DG **Phylodendron** 1990 http://iubio.bio.indiana.edu/treeapp/phylodendron-doc.html

[B22] Cobaleda C Pérez-Caro M Vicente-Dueñas C Sánchez-García I **Function of the zinc-finger transcription factor SNAI2 in cancer and development** Annu Rev Genet 2007 15 41 61 10.1146/annurev.genet.41.110306.130146 17550342

[B23] Capdevila J Tsukui T Esteban CR Zappavigna V Belmonte JCI **Control of vertebrate limb outgrowth by the proximal factor**** *Meis2* **** and distal antagonism of BMPs by gremlin** Mol Cell 1999 15 5 839 849 10.1016/S1097-2765(00)80393-7 10619030

[B24] Dai K Hussain MM **NR2F1 disrupts synergistic activation of the MTTP gene transcription by HNF-4**** *α* **** and HNF-1**** *α* **J Lipid Res 2012 15 5 901 908 10.1194/jlr.M025130 22357705 PMC3329389

[B25] Pioli PD Dahlem TJ Weis JJ Weis JH **Deletion of Snai2 and Snai3 results in impaired physical development compounded by lymphocyte deficiency** PloS one 2013 15 7 69216 10.1371/journal.pone.0069216 PMC3713067 23874916

[B26] Liberg D Sigvardsson M Åkerblad P **The EBF/Olf/Collier family of transcription factors: regulators of differentiation in cells originating from all three embryonal germ layers** Mol Cell Biol 2002 15 24 8389 8397 10.1128/MCB.22.24.8389-8397.2002 12446759 PMC139877

[B27] Bird AD Tan KH Olsson PF Zieba M Flecknoe SJ Liddicoat DR Mollard R Hooper SB Cole TJ **Identification of glucocorticoid-regulated genes that control cell proliferation during murine respiratory development** J Physiol 2007 15 1 187 201 10.1113/jphysiol.2007.136796 17901120 PMC2375468

[B28] Shu W Lu MM Zhang Y Tucker PW Zhou D Morrisey EE **Foxp2 and Foxp1 cooperatively regulate lung and esophagus development** Development 2007 15 10 1991 2000 10.1242/dev.02846 17428829

[B29] Reichardt HM Kaestner KH Tuckermann J Kretz O Wessely O Bock R Gass P Schmid W Herrlich P Angel P etal **Dna binding of the glucocorticoid receptor is not essential for survival** Cell 1998 15 4 531 541 10.1016/S0092-8674(00)81183-6 9604929

[B30] Eden E Navon R Steinfeld I Lipson D Yakhini Z **GOrilla: a tool for discovery and visualization of enriched GO terms in ranked gene lists** BMC Bioinformatics 2009 15 48 10.1186/1471-2105-10-48 19192299 PMC2644678

[B31] Kho AT Bhattacharya S Mecham BH Hong J Kohane IS Mariani TJ **Expression profiles of the mouse lung identify a molecular signature of time-to-birth** Am J Respir Cell Mol Biol 2009 15 1 47 10.1165/rcmb.2008-0048OC 18664640 PMC2606946

[B32] Mariani TJ Reed JJ Shapiro SD **Expression profiling of the developing mouse lung: insights into the establishment of the extracellular matrix** Am J Respir Cell Mol Biol 2002 15 5 541 548 10.1165/ajrcmb.26.5.2001-00080c 11970905

[B33] Mariani TJ Shapiro SD **Application of expression profiling to the developing lung: identification of putative regulatory networks controlling matrix production** Chest 2002 15 3 Suppl 42 44 10.1378/chest.121.3_suppl.42s 11893680

[B34] Chuang P-T McMahon AP **Branching morphogenesis of the lung: new molecular insights into an old problem** Trends Cell Biol 2003 15 2 86 91 10.1016/S0962-8924(02)00031-4 12559759

[B35] Minoo P **Transcriptional regulation of lung development: emergence of specificity** Respir Res 2000 15 2 109 115 10.1186/rr20 11667973 PMC59550

[B36] Weaver M Dunn NR Hogan B **Bmp4 and Fgf10 play opposing roles during lung bud morphogenesis** Development 2000 15 12 2695 2704 10821767 10.1242/dev.127.12.2695

[B37] Perl A-KT Hokuto I Impagnatiello M-A Christofori G Whitsett JA **Temporal effects of sprouty on lung morphogenesis** Dev Biol 2003 15 1 154 168 10.1016/S0012-1606(03)00106-4 12781690

[B38] Chuang P-T Kawcak T McMahon AP **Feedback control of mammalian Hedgehog signaling by the Hedgehog-binding protein, Hip1, modulates Fgf signaling during branching morphogenesis of the lung** Genes Dev 2003 15 3 342 347 10.1101/gad.1026303 12569124 PMC195990

[B39] Bellusci S Furuta Y Rush MG Henderson R Winnier G Hogan B **Involvement of Sonic hedgehog (Shh) in mouse embryonic lung growth and morphogenesis** Development 1997 15 1 53 63 9006067 10.1242/dev.124.1.53

[B40] Weaver M Yingling JM Dunn NR Bellusci S Hogan B **Bmp signaling regulates proximal-distal differentiation of endoderm in mouse lung development** Development 1999 15 18 4005 4015 10457010 10.1242/dev.126.18.4005

[B41] Hyatt BA Shangguan X Shannon JM **BMP4 modulates fibroblast growth factor-mediated induction of proximal and distal lung differentiation in mouse embryonic tracheal epithelium in mesenchyme-free culture** Dev Dyn 2002 15 2 153 165 10.1002/dvdy.10145 12242715

[B42] Li C Xiao J Hormi K Borok Z Minoo P** *Wnt5a* **** participates in distal lung morphogenesis** Dev Biol 2002 15 1 68 81 10.1006/dbio.2002.0729 12142021

[B43] Li C Zhu N-L Tan RC Ballard PL Derynck R Minoo P **Transforming growth factor-**** *β* **** inhibits pulmonary surfactant protein B gene transcription through SMAD3 interactions with NKX2. 1 and HNF-3 transcription factors** J Biol Chem 2002 15 41 38399 38408 10.1074/jbc.M203188200 12161428

[B44] Shi W Heisterkamp N Groffen J Zhao J Warburton D Kaartinen V **Tgf-**** *β* ****3-null mutation does not abrogate fetal lung maturation in vivo by glucocorticoids** Am J Physiol Lung Cell Mol Physiol 1999 15 6 1205 1213 10.1152/ajplung.1999.277.6.L1205 10600892

[B45] Xu Y Wang Y Besnard V Ikegami M Wert SE Heffner C Murray SA Donahue LR Whitsett JA **Transcriptional programs controlling perinatal lung maturation** PloS one 2012 15 8 37046 10.1371/journal.pone.0037046 PMC3423373 22916088

[B46] Cole TJ Solomon NM Van Driel R Monk JA Bird D Richardson SJ Dilley RJ Hooper SB **Altered epithelial cell proportions in the fetal lung of glucocorticoid receptor null mice** Am J Respir Cell Mol Biol 2004 15 5 613 619 10.1165/rcmb.2003-0236OC 14578211

[B47] Gründer A Ebel TT Mallo M Schwarzkopf G Shimizu T Sippel AE Schrewe H **Nuclear factor IB (**** *Nfib* ****) deficient mice have severe lung hypoplasia** Mech Dev 2002 15 1 69 77 11850179 10.1016/s0925-4773(01)00640-2

[B48] Reddy TE Pauli F Sprouse RO Neff NF Newberry KM Garabedian MJ Myers RM **Genomic determination of the glucocorticoid response reveals unexpected mechanisms of gene regulation** Genome Res 2009 15 12 2163 2171 10.1101/gr.097022.109 19801529 PMC2792167

[B49] Polman JAE Welten JE Bosch DS de Jonge RT Balog J van der Maarel SM de Kloet ER Datson NA **A genome-wide signature of glucocorticoid receptor binding in neuronal PC12 cells** BMC Neurosci 2012 15 118 10.1186/1471-2202-13-118 23031785 PMC3519639

[B50] Yu C-Y Mayba O Lee JV Tran J Harris C Speed TP Wang J-C **Genome-wide analysis of glucocorticoid receptor binding regions in adipocytes reveal gene network involved in triglyceride homeostasis** PLoS One 2010 15 12 15188 10.1371/journal.pone.0015188 PMC3004788 21187916

[B51] John S Sabo PJ Thurman RE Sung M-H Biddie SC Johnson TA Hager GL Stamatoyannopoulos JA **Chromatin accessibility pre-determines glucocorticoid receptor binding patterns** Nat Genet 2011 15 3 264 268 10.1038/ng.759 21258342 PMC6386452

[B52] Langmead B Salzberg SL **Fast gapped-read alignment with Bowtie 2** Nat Methods 2012 15 4 357 359 10.1038/nmeth.1923 22388286 PMC3322381

[B53] Zhang Y Liu T Meyer CA Eeckhoute J Johnson DS Bernstein BE Nussbaum C Myers RM Brown M Li W Liu XS **Model-based analysis of ChIP-Seq (MACS)** Genome Biol 2008 15 9 137 10.1186/gb-2008-9-9-r137 PMC2592715 18798982

[B54] Jiang J Chan Y-S Loh Y-H Cai J Tong G-Q Lim C-A Robson P Zhong S Ng H-H **A core Klf circuitry regulates self-renewal of embryonic stem cells** Nat Cell Biol 2008 15 3 353 360 10.1038/ncb1698 18264089

[B55] Wolfe DA Hollander M Nonparametric Statistical Methods 1973 New York: John Wiley

